# Protective Effect of D-Panthenol in Rhabdomyolysis-Induced Acute Kidney Injury

**DOI:** 10.3390/ijms232012273

**Published:** 2022-10-14

**Authors:** Dmitry S. Semenovich, Egor Y. Plotnikov, Elena P. Lukiyenko, Alexander A. Astrowski, Nina P. Kanunnikova

**Affiliations:** 1 A.N. Belozersky Institute of Physico-Chemical Biology, Moscow State University, 119992 Moscow, Russia; 2 Institute of Biochemistry of Biologically Active Substances, NAS of Belarus, 230030 Grodno, Belarus; 3 Faculty of Biology and Ecology, Yanka Kupala State University of Grodno, 230023 Grodno, Belarus

**Keywords:** rhabdomyolysis, acute kidney injury, myoglobin, dexpanthenol, lipid peroxidation, glutathionylation

## Abstract

We investigated the nephroprotective effect of D-panthenol in rhabdomyolysis-induced acute kidney injury (AKI). Adult male Wistar rats were injected with 50% glycerol solution to induce rhabdomyolysis. Animals with rhabdomyolysis were injected with D-panthenol (200 mg/kg) for 7 days. On day 8, we examined AKI markers, renal histology, antioxidant capacity, and protein glutathionylation in kidneys to uncover mechanisms of D-panthenol effects. Rhabdomyolysis kidneys were shown to have pathomorphological alterations (mononuclear infiltration, dilatation of tubules, and hyaline casts in Henle’s loops and collecting ducts). Activities of skeletal muscle damage markers (creatine kinase and lactate dehydrogenase) increased, myoglobinuria was observed, and creatinine, BUN, and pantetheinase activity in serum and urine rose. Signs of oxidative stress in the kidney tissue of rhabdomyolysis rats, increased levels of lipid peroxidation products, and activities of antioxidant enzymes (SOD, catalase, and glutathione peroxidase) were all alleviated by administration of D-panthenol. Its application improved kidney morphology and decreased AKI markers. Mechanisms of D-panthenol’s beneficial effects were associated with an increase in total coenzyme A levels, activity of Krebs cycle enzymes, and attenuation of protein glutathionylation. D-Panthenol protects kidneys from rhabdomyolysis-induced AKI through antioxidant effects, normalization of mitochondrial metabolism, and modulation of glutathione-dependent signaling.

## 1. Introduction

Rhabdomyolysis is a pathology caused by skeletal muscle damage (“crush syndrome”), resulting in the release of muscle myoglobin, sarcoplasmic proteins (creatine kinase, lactate dehydrogenase, aldolase, alanine, and aspartate aminotransferases), and electrolytes into the extracellular space and circulation [1]. Rhabdomyolysis can be caused by various physical or chemical factors such as direct traumatic injury or muscle compression, intense exercise or prolonged immobilization, muscle ischemia, infections, electrolyte and metabolic disorders, genetic diseases, toxins, drugs, and malignant hyperthermia [2,3]. Acute kidney injury (AKI) is the most common life-threatening complication of rhabdomyolysis, with an incidence ranging from 10 to 55% and accompanied by a poor prognosis, especially in the presence of multiple organ failure [4,5]. The development of AKI is considered to be induced primarily by accumulation of myoglobin (possessing nephrotoxic properties) in the kidneys, as well as renal hypoperfusion as a result of systemic hypovolemia. Currently, the treatment of rhabdomyolysis-induced AKI is based on symptomatic and replacement therapy (intravenous fluids, bicarbonate, and hemodialysis), and mortality remains high [6,7].

D-Panthenol (Dexpanthenol) is a precursor of pantothenic acid [8] that has been demonstrated as a nephroprotector in kidney ischemia-reperfusion in rats [9], cisplatin toxicity [10], gentamicin toxicity [11], and sepsis [12]. However, whether D-panthenol could alleviate kidney injury caused by rhabdomyolysis has not been studied.

Considering the mechanisms of rhabdomyolysis-associated AKI, the heme component of myoglobin can stimulate lipid peroxidation due to redox cycling of the heme group from ferrous to ferric and then to ferryl oxidation states, causing renal injury [13]. It is known that an increase in lipid peroxidation in kidney mitochondria and a release of cytochrome c are characteristic of acute glycerol-induced rhabdomyolysis [14]. Mitochondria incubated with myoglobin in vitro have been shown to feature a decrease in respiratory control, uncoupling of oxidative phosphorylation, oxidative damage, and stimulation of NO synthesis [14].

Recently, we have demonstrated that panthenol contributes to the protection of mitochondria through the recovery of energy metabolism, modulation of the redox potential in the glutathione system, and a decrease in the level of S-glutathionylated proteins [15]. Moreover, panthenol affected oxidative stress, mitochondrial metabolism, and Krebs cycle activity [15,16,17,18] that all are affected in AKI. The goal of this study was to explore mechanisms of D-panthenol’s impact on AKI in glycerol-induced rhabdomyolysis.

## 2. Results

### 2.1. Protective Effects on Renal Morphology

**Figure 1:**
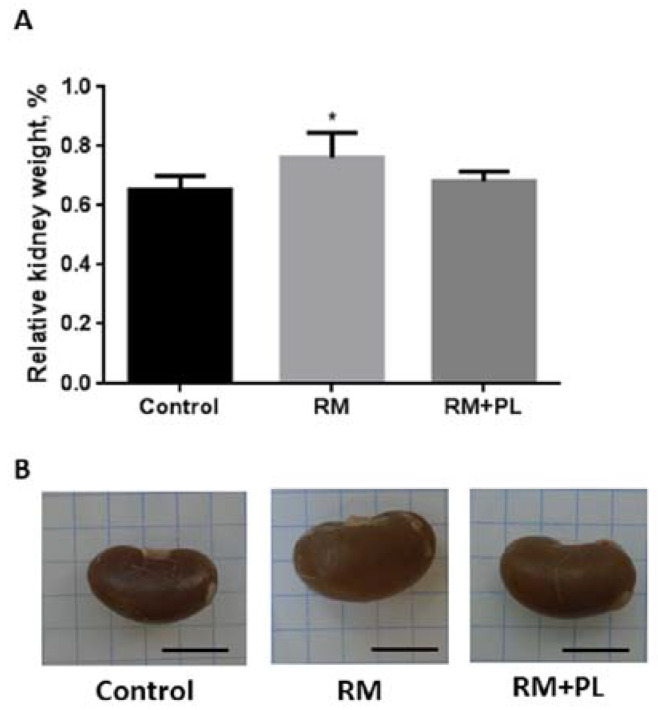
Macromorphological changes in the kidneys 8 days after glycerol-induced rhabdomyolysis and its treatment with D-panthenol: (**A**) changes in the relative weight of rat kidneys (%); and (**B**) rat kidney appearance on the 8th day after rhabdomyolysis. Scale bar, 10 mm. * *p* < 0.05 compared to control.

Glycerol-induced rhabdomyolysis caused severe pathomorphological changes in rat kidneys. We observed a significant increase in the volume of the kidneys and an increase in their weight after rhabdomyolysis (Figure 1). When dissected, the renal medulla tissue in animals was stained dark brown, whereas in PL-treated animals the stain was a light brown color. Such color changes of renal medulla are a conventional characteristic of glycerol-induced rhabdomyolysis associated with the accumulation of myoglobin in the kidney tissue [3]. Injection of RM rats with D-panthenol led to a significant decrease in the volume and weight of the kidneys. Notably, the animals’ body weights did not change during the whole experiment (data not presented).

**Figure 2:**
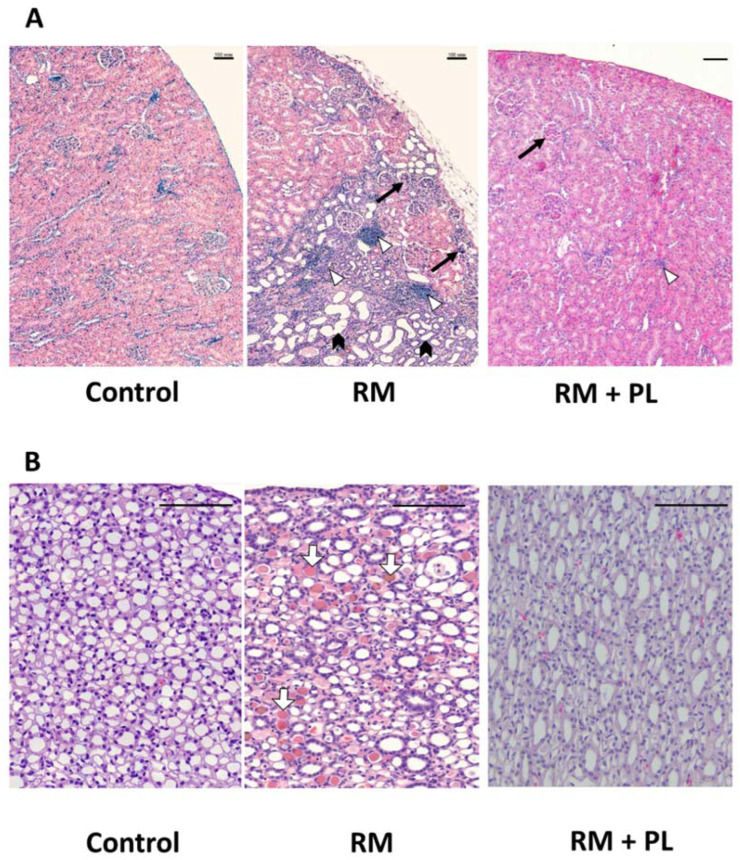
Morphology of the rat kidney cortex in the control group, after rhabdomyolysis (RM) and during rhabdomyolysis with administration of PL (RM + PL). (**A**) Rhabdomyolysis caused pronounced mononuclear infiltration in the cortex (white arrowheads), dilatation of tubules (black arrowheads), glomerular sclerosis (black arrows), and hyaline casts (white arrows). PL reversed these changes. Scale bar, 100 µm. (**B**) The inner part of the kidney medulla. In the RM group, note multiple protein casts (white arrows) in the lumen of Henle loops and collecting ducts. Such masses were incomparably less common after PL treatment.

We also found pathomorphological changes in renal tissue sections stained with hematoxylin-eosin. The loci with diffuse and focal mononuclear infiltration were observed in the kidney cortex after rhabdomyolysis (Figure 2A). The tubular epithelium of some tubules demonstrated more intensive staining with hematoxylin, and the lumen was dilated (less for proximal tubules, and more for distal tubules). Some glomeruli along the kidney capsule were shrunken. In many (especially distal) tubules, as well as in collecting ducts, we detected eosinophilic protein casts (Figure 2A). Loops of Henle and collecting ducts were filled with protein masses (Figure 2B).

**Figure 3:**
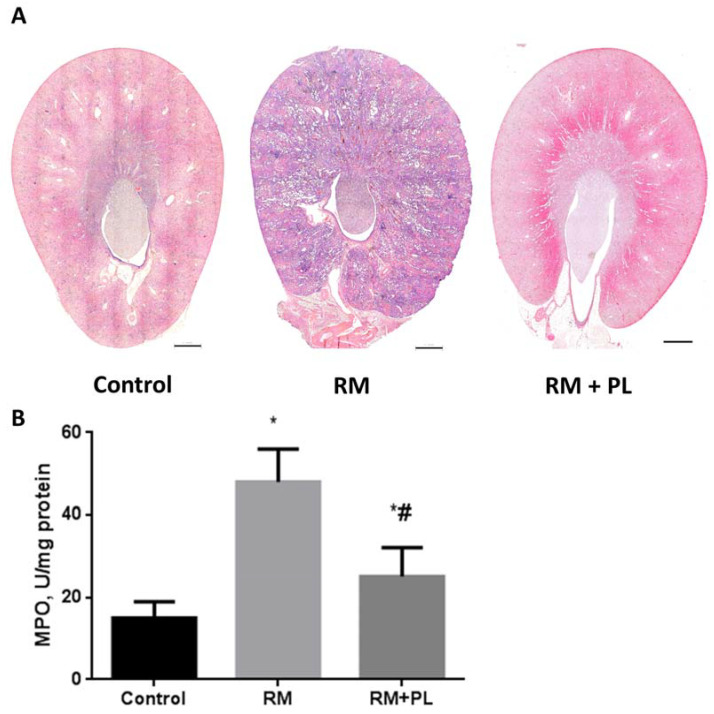
Leukocyte infiltration in the kidney after rhabdomyolysis. (**A**) Panoramic images of transverse sections of the kidneys in the control, rhabdomyolysis (RM), and rhabdomyolysis with PL treatment (RM + PL). Intensive hematoxylin staining of the renal tissue was observed after rhabdomyolysis due to a significantly increased number of nuclei in the infiltrated cell; the PL-group resembled the control. Scale bar, 1 mm. (**B**) Activity of MPO in the kidney tissue during rhabdomyolysis and PL administration. * *p* < 0.05 compared to control, # *p* < 0.05 compared to RM.

Infiltration with leukocytes after RM was observed as an increased number of hematoxylin-positive nuclei in the peri-tubular space of the kidney tissue (Figure 3A), and was then confirmed by determining the activity of MPO in kidney tissue samples (Figure 3B). MPO activity was significantly higher in tissues of the RM group kidneys compared to those of the control, indicating an increased neutrophil infiltration rate compared to control tissue. Further, it was found that PL treatment significantly reduced leukocytes infiltration and the levels of MPO in kidney tissues (Figure 3).

### 2.2. Changes in the Biochemical Parameters of Blood Serum and Urine after Rhabdomyolysis and PL Administration

**Figure 4:**
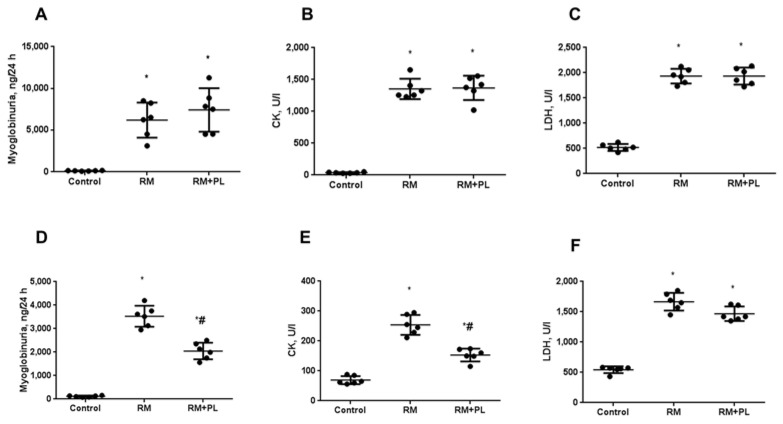
Analysis of muscle damage markers in the urine and serum of rats with rhabdomyolysis and after PL administration. Glycerol-induced rise in urinary myoglobin (**A**), serum CK (**B**), and LDH (**C**) after 24 h. The same parameters after 7 days (**D**–**F**). * *p* < 0.05 compared to control, # *p* < 0.05 compared to RM.

In the first hours after the injection of glycerol, we observed typical signs of rhabdomyolysis well known in clinics for crush syndrome. Thus, a significant myoglobinuria was visually observed as characteristic maple colored urine lasting approximately 48 h after injection. However, this characteristic was subjective and not quantitative, so the breakdown of muscle tissue was confirmed by estimating the activities of serum markers CK and LDH, as well as urinary myoglobin. In RM animals, 24 h after glycerol injection we found a dramatic rise in the activity of CK, LDH, and myoglobin in urine (Figure 4A–C). Administration of PL after rhabdomyolysis did not affect these parameters 24 h after RM induction. Analysis of above-mentioned markers at day 7 after RM revealed a decrease in the severity of muscle tissue damage, as indicated by a decrease in the concentration of myoglobin in urine and a significant decrease in the activities of CK, but not of LDH, in the blood serum. Notably, using PL for 7 consequent days demonstrated some effect on the concentration of markers of muscle damage, as the concentration of myoglobin in urine and CK in serum decreased to a greater extent during treatment with PL (Figure 4D–F).

As a result of rhabdomyolysis and myoglobinuria, AKI developed in rats; thus, the level of NGAL in the urine of RM rats increased 5-fold and the activity of pantetheinase in serum increased 3-fold (Table 1). Furthermore, the development of AKI was estimated using conventional markers, BUN and serum creatinine. Thus, in RM animals, the concentration of urea in the blood serum increased by 2.9 times, and the concentration of creatinine by 2.2 times (Table 1). Administration of PL for 7 days led to a significant decrease in NGAL, pantetheinase, serum creatine, and urea levels, which indicates its nephroprotective effect.

### 2.3. Oxidative Stress and Activity of Antioxidant Enzymes in Renal Tissue during Rhabdomyolysis and PL Administration

**Figure 5:**
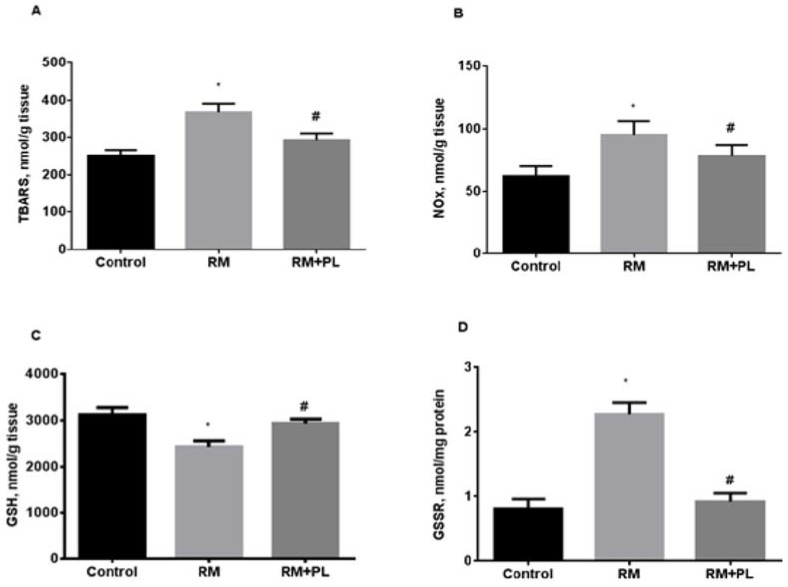
Oxidative stress in the renal tissue during rhabdomyolysis and PL administration: (**A**) levels of TBA-reactive substances (TBARS, nmol/g tissue); (**B**) total nitrates and nitrites (NOx, nmol/g tissue); (**C**) levels of reduced glutathione (GSH, nmol/g tissue); (**D**) levels of mixed protein disulfides with glutathione (GSSR, nmol/mg protein). * *p* < 0.05 compared to control, # *p* < 0.05 compared to RM.

In the kidney tissue of RM rats, the content of lipid peroxidation products and end products of nitric oxide oxidation increased (Figure 5). The levels of TBARS and nitrite/nitrate metabolites increased by 1.5 times in RM animals (Figure 5A,B). The oxidative challenge led to a depletion in the reduced glutathione content, its oxidation, and, finally, to a more than twofold increase in the level of protein disulfides (Figure 5D). Notably, the 7-day administration of PL significantly reduced the level of lipid peroxidation products after rhabdomyolysis. We suggest that PL modulated the thiol-disulfide redox status of the renal tissue and improved kidney tolerance to AKI-induced oxidative damage.

**Figure 6:**
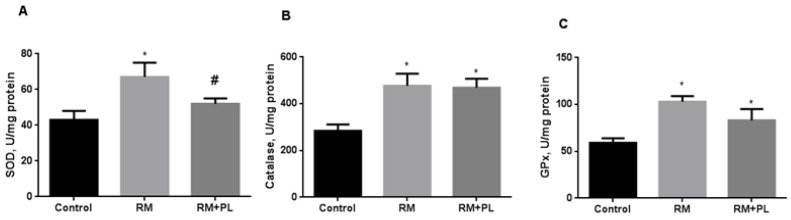
Changes in the activity of antioxidant enzymes in kidney tissue during rhabdomyolysis and PL administration: (**A**) SOD activity; (**B**) catalase activity; and (**C**) glutathione peroxidase activity. * *p* < 0.05 compared to control, # *p* < 0.05 compared to RM.

Activation of free radical processes and accumulation of oxidation products in the renal tissue of RM animals was accompanied by a compensatory induction of antioxidant defense (Figure 6). Catalase and GPx activity increased 1.7 times and SOD activity increased 1.5 times in kidneys from RM rats (Figure 6B,C). Administration of PL to rats with rhabdomyolysis diminished oxidative stress with a subsequent decrease in SOD activity (Figure 6A), whereas catalase and GPx activities remained high (Figure 6B,C).

### 2.4. Determination of Total CoA Levels and Krebs Cycle Enzyme Activity

**Figure 7:**
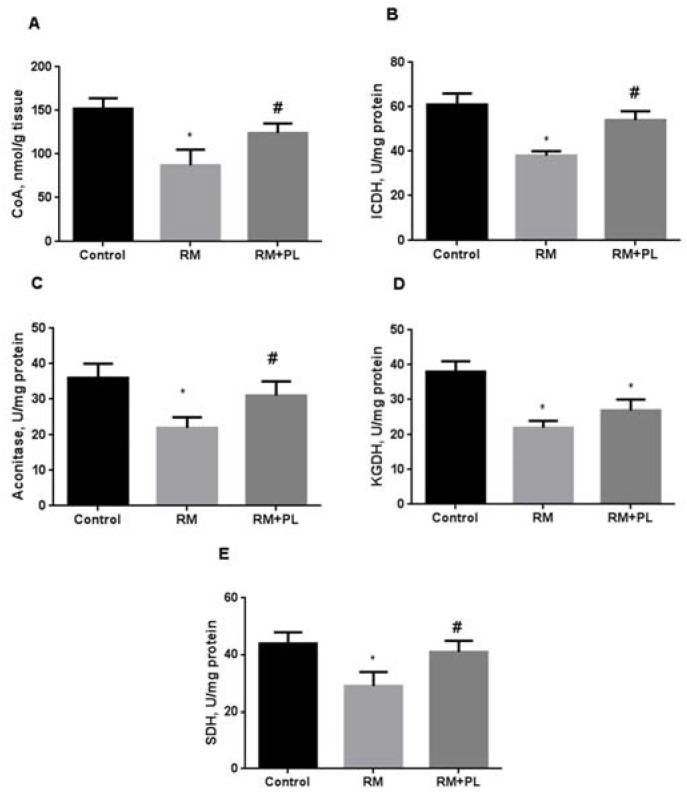
Levels of total CoA in the kidney tissue and the activity of Krebs cycle enzymes in mitochondria of the kidneys after rhabdomyolysis and PL treatment: (**A**) total CoA levels; (**B**) isocitrate dehydrogenase activity; (**C**) aconitase activity; (**D**) alpha-ketoglutarate dehydrogenase activity; and (**E**) succinate dehydrogenase activity. * *p* < 0.05 compared to control, # *p* < 0.05 compared to RM.

In the kidney tissue of RM animals, the level of total CoA decreased by 1.7 times (Figure 7A). We assume that the decrease in the level of CoA was associated with a decrease in total non-protein thiols (as in the case of reduced glutathione) due to the induction of oxidative stress. When PL was administered to RM rats, the CoA levels were restored to values close to those in the control group. Simultaneous with a decrease in the total CoA level, a decrease in the activity of Krebs cycle enzymes was observed in the kidneys of RM animals (Figure 7B–E). As many Krebs cycle enzymes are regulated by glutathionylation, it can be assumed that their activity was suppressed, along with a growth of mixed protein disulfides of glutathione. In addition, we revealed an inhibition of the aconitase activity, probably due to an increase in the level of free iron and/or nitric oxide and its metabolites. Administration of PL to RM rats resulted in an increase in the activity of Krebs cycle enzymes to values close to those in the control group.

## 3. Discussion

D-Panthenol is a derivative of vitamin B5 (pantothenic acid) actively metabolized in mammalian tissues by alcohol dehydrogenase and involved in the biosynthesis of coenzyme A [19,20]. PL is considered to exhibit antioxidant properties, especially by the inhibition of lipid peroxidation in vivo [21,22,23] and in vitro on isolated mitochondria [15,16]. It is well known that glycerol-associated rhabdomyolysis is accompanied by a release of myoglobin into the bloodstream followed by its oxidation and a release of iron ions, which could activate the Fenton reaction and lipid peroxidation in the kidneys [5,6,7]. Thus, the use of PL in RM-induced AKI could be beneficial, as PL directly affects the main pathogenic pathways triggering oxidative stress in renal tissue. Indeed, we found a significant nephroprotective effect of PL. We have shown that using PL during the week after rhabdomyolysis significantly reduced both the severity of acute renal injury and the development of oxidative stress in renal tissue. We have shown earlier that many acute renal pathologies have similar pathogenesis mechanisms and are associated with the development of oxidative stress [24]. Therefore, the use of antioxidant approaches can have a protective effect, ameliorating the damage to renal morphology and function [25,26].

We showed the nephroprotective effect of PL in rhabdomyolysis for the first time; however, the attempt to uncover the mechanisms underlying its antioxidant effects was more important. PL’s impact on the activation of the glutathione redox system and the respiratory activity of Jurkat cells under oxidative stress conditions was demonstrated [27]. We also found that introducing PL after rhabdomyolysis restored the level of reduced glutathione in renal tissue. Our data indicate that this glutathione was replenished from the depot of mixed protein thiols. We believe that this is the most important protective mechanism of PL in acute renal failure; it enables enhanced tolerance of kidney tissue against free radical oxidation. In contrast, enzymatic pathways for replenishing the reduced glutathione pool are unable to synthesize glutathione de novo (in the case of glutathione synthase) or restore it from the disulfide form in the glutathione reductase reaction. Our data indicate that the key pathway of PL influence is associated with a decrease in glutathionylated proteins that was not caused by glutathione reductase stimulation, as PL did not affect its activity.

We observed that the level of free coenzyme A dramatically decreased in renal tissue after rhabdomyolysis, which led to a decrease in the activity of Krebs cycle enzymes. It is known that in the mitochondrial matrix, the content of free coenzyme A can reach a concentration of approximately 1–3 mM, which makes it vulnerable to oxidative damage by free radicals [27,28]. The negative effect of oxidative stress on bioenergetics in kidney cells was effectively reversed by the administration of PL.

An additional aspect of the protective effect of PL may be associated with the suppression of inflammatory processes; we showed a decrease in MPO activity and in leukocyte infiltration in kidney tissue. Similarly, PL was successfully applied in an experimental model of LPS-induced injury, where its anti-inflammatory and antioxidant activity was demonstrated [12]. It should be taken into account that we found some impact of dexpanthenol on muscle recovery after injury, which manifested in a more pronounced decrease in CK and myoglobin in the RM + PL group after a 7-day treatment. However, as the primary muscle damage was the same in both groups at 24 h, which indicates the same myoglobin-induced kidney damage, we could assume that the majority of PL’s nephroprotective effects were associated with direct impact on the kidneys.

**Figure 8:**
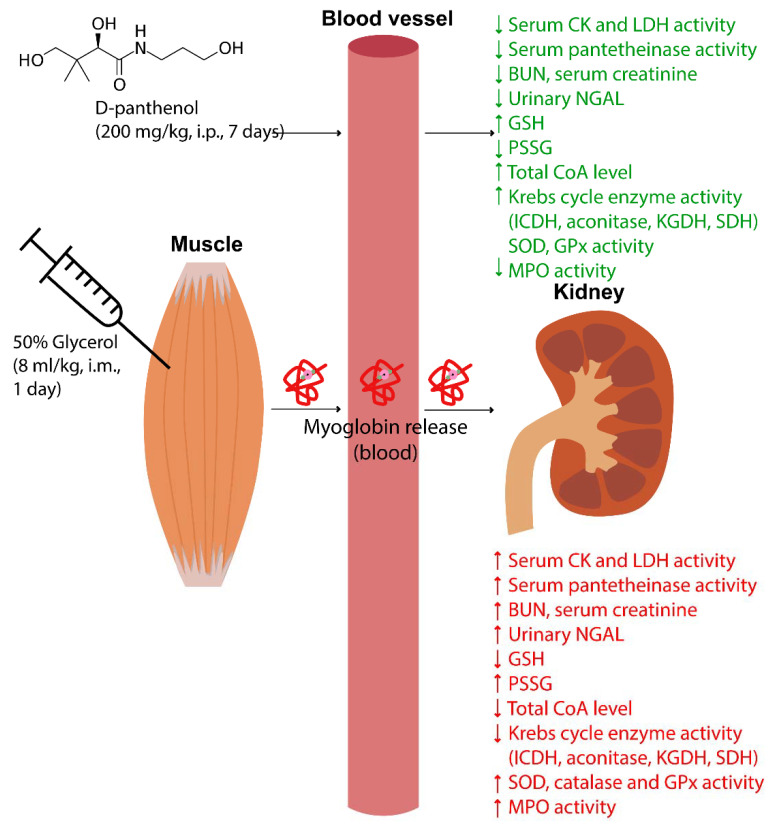
Metabolic effects of dexpanthenol in glycerol-induced rhabdomyolysis and acute renal failure in rats.

The possible mechanisms of PL-induced nephroprotection under glycerol-induced rhabdomyolysis are summarized in Figure 8.

## 4. Materials and Methods

### 4.1. Chemicals

Reagents were obtained as follows: Tris base, tert-butyl hydroperoxide (TBHP), coenzyme A (CoA) lithium salt, sucrose, β-nicotinamide adenine dinucleotide phosphate reduced (NADPH), glutathione reductase from baker’s yeast, D-panthenol (PL), sodium 2-oxoglutarate, sodium succinate, and sodium azide, from Sigma-Aldrich (Merck, Darmstadt, Germany); and 2-thiobarbituric acid, reduced and oxidized glutathione, 5,5′-dithio-bis-(2-nitrobenzoic acid) (DTNB), trichloroacetic acid (TCA), and Folin–Ciocalteu’s reagent, from AppliChem GmbH (Darmstadt, Germany). All reagents were of analytical grade, HPLC grade, or the best available pharmaceutical grade. All solutions were prepared using water purified by the Milli-Q system.

### 4.2. Animals

Experiments were carried out on 6-month-old male Wistar rats weighing 300–350 g (*n* = 18) kept under standard vivarium conditions. The animals had unlimited access to food and water and were kept in cages in a temperature-controlled environment (20 ± 2 °C) with a 12/12-h light/dark regime. Experiments were carried out in accordance with the ethical standards and recommendations for the maintenance and care of laboratory animals provided by the Directives of the Council of the European Community 2010/63/EU on the use of animals for experimental research. Experiments were approved by the Ethics Committee of the Institute of Biochemistry of Biologically Active Substances (protocol 1/17 dated 6 February 2017). Animals were humanely euthanized in accordance with accepted bioethical requirements.

### 4.3. Experimental Design

Rats (*n* = 18) were divided into 3 groups of 6 animals each: control animals; animals with rhabdomyolysis (RM); and RM animals treated with D-panthenol (RM + PL). For the induction of rhabdomyolysis in rats, a 50% aqueous solution of glycerol was injected into the femoral muscles of both hind limbs at half a dose (8 mL/kg, once) [29]. Control animals were intramuscularly injected with saline (8 mL/kg, once). After the injection of glycerol, animals in the RM + PL group were intraperitoneally injected with PL (200 mg/kg) 1 h post-glycerol and daily for 7 consecutive days. Control group animals received saline intraperitoneally. One day prior to euthanasia, animals were placed in metabolic cages to collect urine for 24 h. Daily urine was placed in containers, centrifuged at 3000 rpm for 10 min and stored at −20 °C. Whole blood and kidney tissue were collected on day 8. Serum was stored at −20 °C. Right kidneys were used for histological study. Left kidneys were placed in a cold isolation medium and used to isolate mitochondria.

### 4.4. Morphological Studies of Kidneys

Kidneys were removed, placed on glass slides, weighed and photographed. Relative kidney weights were calculated as a ratio of the weight of the kidneys to body weight (in %) [30].

Right kidneys were fixed for 3 h in a mixture of 37% formalin, 95% ethanol, and glacial acetic acid in a volume ratio of 9:3:1, washed with running tap water, and embedded in paraffin according to the conventional protocol. Next, transverse kidney sections 5 μm thick were obtained. Sections were stained with hematoxylin and eosin and photographed using a Leica DM 1000 microscope with 4× and 20× objectives.

### 4.5. Biochemical Parameters of Blood Serum and Urine

Muscle damage was assessed via the activity of creatine kinase (CK) and lactate dehydrogenase (LDH) in blood serum and the development of myoglobinuria. The development of AKI was assessed via levels of creatinine, BUN in serum, as well as changes in the activity of pantetheinase and NGAL levels.

The activities of CK and LDH in serum were determined using the kinetic method on a SOLAR PV1251C semi-automatic analyzer (SOLAR, Minsk, Belarus) using reagent kits from Analiz-X (Minsk, Belarus).

Urinary levels of NGAL and myoglobin were determined using ELISA according to the manufacturer’s protocol (FineTest, Wuhan, China).

Levels of serum creatinine were determined using a kinetic method based on the Jaffe reaction [31] by absorption of a complex of picrate with creatinine in an alkaline medium at 490 nm detected using a SOLAR PB2201C spectrophotometer (SOLAR).

BUN levels were determined using the diacetyl monooxime method [32] by absorption of the colored complex at 540 nm on a SOLAR PB2201C spectrophotometer.

The activity of pantetheinase in blood serum was determined using the spectrophotometric method by absorption of p-nitroaniline at 405 nm, formed during the hydrolysis of pantothenate-p-nitroanilide [33]. Absorption was measured using a Vityaz F300TP photometer (Vityaz, Vitebsk, Belarus).

### 4.6. Kidney Homogenization and Isolation of Mitochondria

Left kidneys were removed, weighed and homogenized in a cold isolation medium containing 250 mM sucrose, 10 mM Tris HCl and 1 mM EDTA, with a pH of 7.4 at a ratio of 1:10 (*w*/*v*). Mitochondria were isolated using differential centrifugation [34]. The homogenate was centrifuged at 800× *g* for 10 min at 4 °C. The supernatant was centrifuged at 8500× *g* for 10 min at 4 °C. Mitochondrial pellets were washed twice with the isolation medium and resuspended in a hypotonic buffer (10 mM Tris HCl, 0.5 mM EDTA) for a protein concentration of 15–20 mg/mL. Concentrations of total protein were determined using the G. L. Peterson method [35]. To determine the activity of Krebs cycle enzymes, mitochondria were subjected to three freeze–thaw cycles.

### 4.7. Oxidative Stress, MPO, and Antioxidant Enzyme Activity Estimation

TBARS was determined using the H. Ohkawa method [36] by absorption of a complex of thiobarbituric acid with malondialdehyde in an acid medium at 532 nm.

The total level of nitrite/nitrate (NOx) was estimated in a Griess reaction detected by absorption at 540 nm. To reduce nitrates, cadmium granules activated with copper sulfate were used [37].

Reduced glutathione (GSH) was determined in protein-free kidney tissue extracts treated with 0.5 M perchloric acid neutralized by 5 M potassium carbonate to pH 6–7. GSH was determined in a color reaction with DTNB according to the Ellman method [38]. Briefly, the tissue extract was mixed with a 0.1 M potassium phosphate buffer, pH 8.0, containing 5 mM EDTA and 10 mM DTNB in methanol; absorption was determined at 412 nm.

Mixed disulfides of proteins (GSSR) with glutathione were determined by reducing the disulfide groups of tissue proteins with sodium borohydride [39] and determining the released glutathione in the reaction with DTNB [38].

MPO activity was determined using the reaction of hypochlorous acid with taurine. Chloramine-taurine reacts with 5-thio-2-nitrobenzoic acid (TNB) resulting in reduced absorption at 412 nm [40]. The molar absorption coefficient of TNB was 14.150 mM^−1^cm^−1^.

The SOD activity was determined using an indirect spectrophotometric method based on inhibition of the quercetin autooxidation reaction by SOD in an alkaline medium at 405 nm [41].

The catalase activity was determined using a colorimetric method based on the reaction of hydrogen peroxide with ammonium heptamolybdate, which forms a complex with absorbance at 405 nm [42].

The activity of glutathione peroxidase (GPx) was determined using a kinetic method based on NADPH consumption by glutathione reductase in the reaction with oxidized glutathione [43]; tert-Butyl hydroperoxide was used as a GPx substrate.

### 4.8. Determination of Total CoA and Krebs Cycle Enzyme Activity

The determination of total coenzyme A (CoA) was carried out using a spectrofluorimetric method based on the alpha-ketoglutarate dehydrogenase reaction [44]. The enzyme was isolated from rat hearts using ammonium acetate fractionation and gel filtration [45]. For analysis, kidney tissue was homogenized in 0.25 M KOH in ethanol and incubated with dithiothreitol at 60 °C for 30 min. Samples were cooled in ice and neutralized to pH 5–6 with 0.5 M triethanolamine HCl containing 0.6 M perchloric acid, and centrifuged for 15 min at 12,500 rpm. The supernatant was used for analysis.

To determine the activity of isocitrate dehydrogenase (ICDH), we used a reaction solution of 0.05 M Tris-HCl (pH 7.4), 0.2 mM MnCl_2_, 0.4 mM NADP, and 0.05 mM sodium isocitrate [46]; the activity of aconitase was determined in 0.1 M Tris-HCl (pH 7.4), 0.6 mM MnCl_2_, 0.2 mM NADP, 1.0 mM sodium citrate, and 1.0 U isocitrate dehydrogenase [47]. The change in absorbance at 340 nm was determined, and the activity of ICDH and aconitase was calculated using a molar extinction coefficient of 6.22 mM^−1^cm^−1^.

The activities of succinate dehydrogenase (SDH) and alpha-ketoglutarate dehydrogenase (KGDH) were assessed using potassium ferricyanide as described in [48,49]. The activity of KGDH was determined in a reaction medium containing a 50 mM potassium phosphate buffer (pH 7.4), 1 mM MgCl_2_, 3 mM sodium 2-oxoglutarate, 0.2 mM thiamine diphosphate, and 0.6 mM potassium ferricyanide. SDH activity was determined in a reaction medium containing a 100 mM potassium phosphate buffer (pH 7.4), 10 mM sodium succinate, 2.5 mM EDTA, 15 mM sodium azide, and 2 mM potassium ferricyanide. The reaction was stopped after 30 min by adding cold 10% TCA. Samples were centrifuged for 5 min at 6000 rpm, and residual ferricyanide was determined at 420 nm.

### 4.9. Statistical Analysis

Statistical analysis was performed using Microsoft Excel 2016 and GraphPad Prism 6.0. Data are presented as means ± SEM. The significance was assessed using one-way ANOVA with Tukey’s test. In all cases, differences were considered statistically significant at *p* < 0.05.

## 5. Conclusions

We proved the efficacy of D-panthenol in the treatment of rhabdomyolysis-induced AKI and identified the key mechanisms of PL-mediated renoprotection. Our findings underline the role of the glutathione redox system in the damage and protection of kidney tissue, indicating possible targets for further studies of PL and related substances in acute kidney injury.

## Figures and Tables

**Table 1:** Levels of NGAL in urine, activity of pantetheinase and concentration of creatinine and BUN in blood serum of rats at 7 day after rhabdomyolysis and after 7 days of PL administration.

Groups	Urinary NGAL, ng/24 h	Serum Pantetheinase, U/L	Serum Creatinine, µmol/L	BUN, mmol/L
Control	201 ± 40	16 ± 4	65 ± 6	6.4 ± 1.2
RM	2220 ± 440 *	52 ± 8 *	142 ± 10 *	18.5 ± 1.5 *
RM + PL	1258 ± 230 *#	31 ± 6 *#	106 ± 8 *#	10.3 ± 1.4 *#

Note: * *p* < 0.05 compared to control, # *p* < 0.05 compared to RM.

## Data Availability

Data generated and analyzed during the current study are available from the corresponding author on reasonable request.
